# Adverse Childhood Events are Associated with Poor Mental Health in Young, Newly Married Women in Nepal

**DOI:** 10.1177/08862605261446978

**Published:** 2026-05-31

**Authors:** Alison M. El Ayadi, Saira Khan, Mahesh C. Puri, Nadia G. Diamond-Smith

**Affiliations:** 1 University of California, San Francisco, USA; 2 Independent Consultant, New York City, NY, USA; 3 Center for Research on Environment, Kathmandu, Nepal

**Keywords:** adverse childhood experiences, child maltreatment, gender-based violence, health effects, intimate partner violence abuse, mental health and violence

## Abstract

Adverse childhood events are associated with short- and long-term physical and mental health outcomes, as well as risk of future continued exposure to violence. There is limited research on adverse childhood events in lower resource settings and its impact on mental health among women. Using data from 200 newly married women living in rural Nepal, we explored the impact of adverse childhood events on depressive symptoms and intimate partner violence (IPV) in early adulthood. Most study participants reported at least one adverse childhood experience (ACE) (58.5%), one-quarter depressive symptoms (24.5%), and one-quarter (24.5%) reported any IPV. Each ACE reported was associated with a mean 1.92-point increase in depressive symptom score (95% confidence interval [CI] [1.11, 2.73]) and a mean 0.23-point (95% CI [0.20, 0.36]) increase in severity of IPV. IPV was identified as an independent contributor to but not a significant mediator of depressive symptoms. These findings suggest that ACEs are an important influencer of young adult poor mental health, particularly among women, and inform the unique pathways through which distal and proximate factors such as these influence mental health status. Policies and programs are needed not only to prevent and mitigate the impact of ACEs in the natal home, but also to target the marital home and the transition to marriage as key opportunities to build safe spaces and support for young married women in Nepal and similar contexts.

## Introduction

Adverse childhood experiences (ACEs), stressful or traumatic events during childhood such as physical and emotional abuse or neglect, are associated with a range of adverse impacts across the life course (Felitti, 2002; Felitti et al., 1998; Lynch & Smith, 2005). Exposure to ACEs increases the likelihood of engaging in health-risk behaviors and experiencing adverse health outcomes both in the short-term and in the long-term (Anda et al., 2006; Hughes et al., 2017). For example, children and adolescents who report ACEs have higher rates of asthma and allergies, depression and self-harm (Björkenstam et al., 2015; Exley et al., 2015; Oh et al., 2018; Schilling et al., 2007). Similarly, adults who report ACEs are at higher risk of substance use and abuse, inflammatory diseases, obesity, diabetes, violence victimization, and poor mental health outcomes, including psychiatric disorders and suicidal ideation (Afifi et al., 2008; Bellis et al., 2015; Douglas et al., 2010; Hillis et al., 2000; Hughes et al., 2017; Rehkopf et al., 2016).

The relationship between ACEs and long-term negative health outcomes is often explained by allostatic load theory, which posits that chronic or significant stressors during childhood impair neurobiological, endocrine, and immune system development, resulting in biological and behavioral dysregulation and negative psychological coping strategies. These disruptions increase later life risk of chronic disease and psychiatric disorders (Beckie, 2012; Dickie et al., 2024; McEwen, 2004; Sheffler et al., 2020). ACE-related disruptions in adaptive emotional regulation development demonstrate independent and interactive psychosocial, behavioral, and biological impacts, influencing life trajectories and increasing vulnerability to revictimization, including intimate partner violence (IPV; Walker & Wamser-Nanney, 2023). Studies have identified a dose–response inverse relationship between ACE exposure and later life health for both infectious and non-communicable diseases (Anda et al., 2006). However, protective factors, such as growing up with an adult who provides feelings of safety and protection and overall exposure to safe, stable, and nurturing relationships, can moderate these impacts (Crouch et al., 2019).

The link between ACEs and mental health morbidity is well established in high-income countries (Atzl et al., 2019; De Venter et al., 2013; Jones et al., 2018; Raposo et al., 2014; Sareen et al., 2013; Schilling et al., 2007; Sheffler et al., 2020; Wiens et al., 2020). ACE exposure has been associated with antisocial behavior and substance use among young adults (Schilling et al., 2007) and an increased risk for mental health disorders during adolescence (Rytilä-Manninen et al., 2014) and adulthood (Sheffler et al., 2020, p. 202). Additionally, ACEs are strongly associated with later life violence exposure (Lee et al., 2020; Mair et al., 2012), including IPV (Capaldi et al., 2012). In high-income settings, cross-sectional studies suggest that ACEs contribute to IPV directly and indirectly and directly through factors like depression, anxiety, impulsivity, and alcohol use (Lee et al., 2020; Mair et al., 2012). Longitudinal studies support a temporal relationship between IPV and depression, with potential differences in directionality (Watson & Bitsika, 2025). Childhood experiences of physical and sexual violence are associated with revictimization or perpetration later in life (Capaldi et al., 2012). The impact of ACEs extends beyond cognitive, social, and emotional impairments in childhood or young adulthood (Iob et al., 2021; Mersky et al., 2013; Scorza et al., 2022), to adult mental health and its consequences, including anxiety (Green et al., 2010), depression (Poole et al., 2017), low educational attainment, unemployment, and poverty (Metzler et al., 2017).

While research has largely focused on high-income settings, emerging evidence suggests that the relationship between ACEs and mental health outcomes is similar in low- and lower-middle-income-countries (LMICs) (Fulu et al., 2013; Schilling et al., 2007). However, LMIC settings may have greater prevalence of limited resources and poor social protection, both important ACE risk factors (Almuneef et al., 2014; Dorji et al., 2020; Ramiro et al., 2010). For example, a study in the Philippines found that 75% of adults had experienced at least one ACE and 9% had experienced four or more (Ramiro et al., 2010). These exposures were associated with higher rates of smoking, alcohol use, early sexual activity, sex with multiple sexual partners, adolescent pregnancy, and unintended pregnancy (Ramiro et al., 2010). Similarly, in Sri Lanka, men with ACE exposure were more likely to perpetrate IPV, including emotional, financial, physical, and sexual violence (Fonseka et al., 2015). This limited evidence suggests that ACEs are an important public health problem in LMICs, supporting the need for further investigation into prevention and intervention strategies to mitigate ACEs-related morbidity throughout the life course.

One critical existing gap on ACEs in LMICs is the intersection of gender equality and ACE exposure. For example, in Nepal, mental health disorders are prevalent among young and postpartum women (Bhusal et al., 2016; Budhathoki et al., 2012; Chalise & Bhandari, 2019; Giri et al., 2015; Khadka et al., 2019; Kunwar et al., 2015; Regmi et al., 2002; Singh et al., 2021; Subba et al., 2013). Gender-specific factors such as stressful life conditions, marital dynamics (e.g., love vs. arranged marriages), polygamy, and IPV have been linked to depressive symptoms among postpartum women (Ho-Yen et al., 2007; Kohrt et al., 2009) and women of reproductive age (Budhathoki et al., 2012; Clark et al., 2019; Koirala & Chuemchit, 2020). Nepal’s patriarchal family structure, including patrilocality, where married couples typically reside with the husband’s parents, further limits women’s decision-making power regarding relationships and reproduction (Diamond-Smith, Plaza, et al., 2020; Puri et al., 2010). This dynamic may exacerbate ACE-related impacts, particularly among young, newly married women who hold the lowest status in their household. These women are more likely to experience IPV (Raifman et al., 2021), and lower household status has been associated with greater depressive symptom severity (Gopalakrishnan et al., 2023b).

Given the unique sociocultural factors in this setting and existing evidence gaps, our study sought to explore the association between ACE exposure in childhood, IPV after marriage, and mental health outcomes among young (<25 years old), newly married women in Nepal. Specifically, we investigated how ACE severity influences depressive symptoms shortly after transitioning from their childhood homes to their husbands’ households, hypothesizing that ACEs exposure severity directly impacts mental health and increase likelihood of violence revictimization through IPV, directly and indirectly influencing mental health outcomes.

## Methods

### Study Design and Participants

These analyses used data from a longitudinal study on the health of newly married women in Nawalparasi district, Nepal, located on the border with India. Data were collected from February 2018 to August 2020 at 6-month intervals. The current analysis uses data only from the first round of data collection (2018), when women had recently moved into their marital home [Diamond-Smith, Shieh, et al., 2020]. Briefly, data were collected from 200 women aged 18 to 25, currently living with their mother-in-law and recently married. We recruited participants from a sampling frame of newly married women who were identified in one rural municipality and one urban municipality through household mapping via assistance from community leaders, such as female community health volunteers, teachers, health workers, and religious leaders. A total of 302 eligible women were identified and 200 women (100 urban, 100 rural) were selected randomly. Potential participants were approached in their homes by one of four trained female researchers. Women underwent an informed consent process and were interviewed in a private location if they consented, usually their home or a nearby field. Where appropriate, permission was also asked from the women’s family/husband, given cultural norms in the study area. Two women were not allowed to participate in the study by their family members and were replaced by the nearest eligible women in the same area.

### Study Measures

Study participants self-reported sociodemographic characteristics, relationship quality, mental health status, IPV, and exposure to ACEs. Sociodemographic characteristics included age (continuous), educational attainment (continuous years of schooling classified into categories: none, primary 1 to 8, secondary 9 to 12, greater than 12, in alignment with the Nepal Demographic and Health Survey), household wealth in quartiles (compiled from indicator variables on household ownership of select household assets, housing materials, access to water and sanitation facilities, and regular household cooking fuels using principal components analysis and classified into quartiles, in alignment with the Nepal Demographic and Health Survey), (Ministry of Health and Population et al., 2023) and time since marriage (in months). Relationship quality with husband was assessed through the spousal relationship quality scale, which evaluates three dimensions of commitment, trust, and satisfaction through 21 items using likert-style response categories reflecting agreement with a statement (strongly disagree, disagree, agree, strongly agree, 0–3) (Gopalakrishnan et al., 2023a). The items were summed (range 0–63) and categorized by quartile into four levels reflecting low (<16), medium (16–31), high (32–47), and highest (48–63) relationship quality. Relationship quality with mother-in-law was assessed similarly through a subset of seven items adapted from the spousal relationship quality scale, which were summed (range 0–21) and categorized by quartile into four levels reflecting low (<5), medium (5–9), high (10–14), and highest (15≤) relationship quality (Gopalakrishnan et al., 2023a). Mental health status was assessed using the Hopkins Symptoms Checklist, which has been previously validated for use in Nepal (Thapa & Hauff, 2012), and operationalized continuously using the mean value (range 1–4) and using a cutoff of 1.75 to represent probable depression (Sandanger et al., 1998). Exposure to IPV by the current partner was collected using an instrument previously tested in Nepal, which included 17 questions on psychological violence, physical violence (any pushed/shaken, threw something, slapped, twisted arm/pulled, punch/hurt, kicked/dragged/beat, choked or burned, threatening or attacking with a weapon), economic control (any prohibiting from working/training/earning money, took earnings or property against will, threw out of house), threatening children, and sexual violence (any forced sex or sexual acts, withholding things for sex)(Raifman et al., 2021). IPV severity score was calculated from the sum of IPV questions (range 0–17), and operationalized as a continuous score, ever IPV overall and ever IPV, by domain. Exposure to ACEs that occurred under the age 18 in the participant’s natal home was collected through the WHO ACEs International Questionnaire (ACE-IQ), which assesses family dysfunction; physical, sexual, and emotional abuse and neglect by parents or caregivers; peer violence; witnessing community violence; and exposure to collective violence (World Health Organization, 2018). We included a subset of 12 questions from ACE-IQ domains: parental presence ( alcohol/drug abuser in household; one or no parents, parental separation or divorce); household violence (witnessed household member treated violently, witnessed household member being verbally abused); emotional abuse and neglect (parent/guardian withheld food from respondent, experienced verbal abuse from parent/household member, parent/guardian threatened to abandon respondent or actually abandoned child), physical abuse (parent/guardian physically abused respondent, parent/guardian physically abused respondent with object), contact sexual abuse (sexually touched without consent, forced to sexually touch without consent, attempted or forced oral, anal, or vaginal intercourse without consent). Questions on parents’ mental health and parent in jail were removed based on in-country partner recommendation. ACEs score was calculated as the number of ACEs endorsed by the participant (range 0–12). Internal consistency reliability for all measures ranged from good to high within this sample (husband relationship quality, α = .82; mother-in-law relationship quality, α = .90; depressive symptoms, α = .93; and severity of IPV, α = 0.84).

### Conceptual Framework and Data Analysis

**Figure 1.**
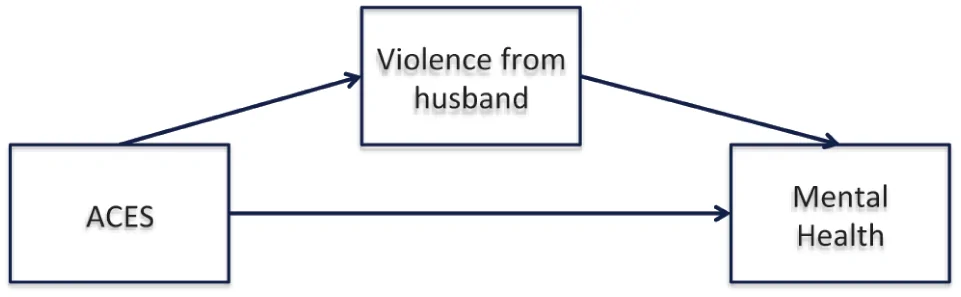
Summary of conceptual framework between ACEs exposure, intimate partner violence and depressive symptoms. ACE: Adverse childhood experience.

Our summarized conceptual framework postulates direct and indirect pathways between ACEs in childhood and current mental health, with IPV operating as a partial mediator between ACEs and mental health (Figure 1). Participant sociodemographic characteristics, exposure to ACEs, mental health, and IPV were described using means and standard deviations (*SD*s), medians and interquartile ranges, and proportions. We employed structural equation modeling to estimate relationship between ACEs on depressive symptoms and examined the extent to which IPV mediates this association (Gunzler et al., 2013). We included sociodemographic characteristics specified a priori (i.e., age group, educational attainment, wealth quintile, and rurality), and quality of relationship with husband. We decomposed effects into direct, indirect, and total using Sobel’s method, and obtained bias-corrected 95% confidence intervals (*CI*s) using the bootstrapped delta method (Sobel, 1987). Analyses were conducted using Stata v. 17 (College Station, TX), and differences were considered statistically significant where *p* < .05.

### Ethical Approval

All study procedures were reviewed and approved by the Nepal Health Research Council and the Institutional Review Board of the University of California San Francisco. All participants provided written confirmation of informed consent.

## Results

### Sociodemographic Characteristics of Study Participants

Mean study participant age was 20.4 (*SD* 2.0; Table 1). Most had received some schooling: 27% 1 to 8 years, 54% 9 to 12 years, and 15% over 12 years. The majority (86%) were Hindu, 8.5% Muslim, 4.5% Buddhist, and 1% other (Bon or Christian). Most reported their marriage was arranged (70.5%).

**Table 1.** Sociodemographic Characteristics of Newly Married Women Study Participants, Nawalparasi, Nepal, 2018 (*n* = 200).

Characteristic	*N*	%
Age in years^a^	20.4 (2.0)	
Educational attainment		
None	8	4.0
Primary (1–8)	54	27.0
Secondary (9–12)	108	54.0
More than secondary (>12)	30	15.0
Type of marriage		
Arranged	141	70.5
Love	59	29.5
Religion		
Hindu	172	86.0
Muslim	17	8.5
Buddhist	9	4.5
Other	2	1.0
Caste/ethnic group		
Terai Janjati	78	39.0
Brahmin	30	15.0
Janajati	24	12.0
Tarai Dalit	18	9.0
Muslim	16	8.0
Chhetri	11	5.5
Other	23	11.5
Has been pregnant before	17	8.5
Has toilet and running water	54	27.0
Has electricity	199	99.5
Main type of fuel household uses for cooking		
LPG gas	99	49.5
Firewood	90	45.0
Other	11	5.5
Parents live in same community	29	14.5
Wealth quintile		
Lowest	40	20.0
Second	40	20.0
Middle	40	20.0
Fourth	40	20.0
Highest	40	20.0
Municipality type		
Rural	100	50.0
Urban	100	50.0
Relationship quality–husband		
Lowest	0	0.0
High	11	5.5
Highest	189	94.5

a Mean (*SD*).

### Experience of ACEs

Most study participants reported at least 1 ACE (58.5%; Table 2). Median number of ACEs was 1.0 (Interquartile range [IQR] 1.0–2.0). The most common ACEs domains reported were detrimental effects of parental presence (37.5%), emotional abuse and neglect (31.5%), and household violence (27.0%). Fewer study participants reported physical abuse (10.0%) or sexual abuse (2.0%). More frequent ACEs exposure was reported for household violence and emotional abuse and neglect domains, where key experiences included verbal abuse from a parent or household member (31.0%), a parent or guardian who was an alcoholic or abused drugs (28.5%) or having witnessed a parent or household member being verbally abused (25.5%). Most individuals endorsing these ACEs reported that they occurred ‘a few times’.

**Table 2.** Frequency of Exposure to ACEs Overall and by Domain, Newly Married Women Study Participants, Nawalparasi, Nepal, 2018 (*n* = 200).

ACEs overall and ACE domain	Any exposure	Frequency of exposure			
	*N* = 200	Many times	A few times	Once	Never
	*n* (%)	*n* (%)	*n* (%)	*n* (%)	*n* (%)
ACEs	117 (58.5)				
Number of ACEs					
Median (interquartile range)	1.0 (0.0–2.0)				
Min, max	0, 8				
Number of ACE domains					
Median (interquartile range)	1.0 (0.0–2.0)				
Min, max	0, 5				
ACEs domains					
*Parental presence*	**75 (37.5)**				
Parent/guardian alcoholic/drug abuser	57 (28.5)				
Death of parent/guardian	26 (13.0)				
*Household violence*	**54 (27.0)**				
Witnessed parent/household member being verbally abused	51 (25.5)	3 (1.5)	38 (19.0)	10 (5.0)	149 (74.5)
Witnessed parent/household member being physically abused	18 (9.0)	0 (0.0)	10 (5.0)	8 (4.0)	182 (91.0)
*Emotional abuse and neglect*	**63 (31.5)**				
Parent/guardian withheld food from respondent (*n* = 199)	4 (2.0)	0 (0.0)	3 (1.5)	1 (0.5)	195 (98.0)
Experienced verbal abuse from parent/household member	62 (31.0)	3 (1.5)	51 (25.5)	8 (4.0)	138 (69.0)
Parent/guardian threatened/actually abandoned	9 (4.5)	0 (0.0)	6 (3.0)	3 (1.5)	191 (95.5)
*Physical abuse*	**20 (10.0)**				
Parent/guardian physically abused respondent	20 (10.0)	0 (0.0)	15 (7.5)	5 (2.5)	180 (90.0)
Parent/guardian physically abused respondent with object	5 (2.5)	0 (0.0)	1 (0.5)	4 (2.0)	195 (97.5)
*Sexual abuse*	**4 (2.0)**				
Sexually touched without consent	3 (1.5)	0 (0.0)	1 (0.5)	2 (1.0)	197 (98.5)
Forced to sexually touch without consent	3 (1.5)	0 (0.0)	0 (0.0)	3 (1.5)	197 (98.5)
Attempted or forced oral, anal or vaginal intercourse without consent	2 (1.0)	0 (0.0)	0 (0.0)	2 (1.0)	198 (99.0)

*Note.* ACE = Adverse childhood experience.

The distribution of ACEs overall and by domain was patterned significantly by age, marriage type, caste, prior pregnancy status, educational attainment, rural/urban residence, parental residence, and mother-in-law relationship quality (Tables S1a and S1b), with some variation by domain.

### Mental Health

Across participants, the median depressive symptoms score was 1.2 (IQR 1.0–2.7; Table 3). One-quarter (24.5%) had depressive symptom score above the threshold, suggesting major depressive disorder.

**Table 3.** Mental Health Status and Exposure to Intimate Partner Violence by Domain, Newly Married Women Study Participants, Nawalparasi, Nepal, 2018 (*n* = 200).

Participant health status and exposure	*n* (%)
Depressive symptoms	
Depressive symptoms (median, interquartile range)	1.2 (1.0–1.7)
Severity of symptoms suggestive of major depressive disorder	49 (24.5)
Intimate partner violence	
Any violence across all domains	49 (24.5)
Violence by domain	
Psychological violence	12 (6.0)
Physical violence	7 (3.5)
Economic violence	25 (12.5)
Sexual violence	32 (16.0)

### Experience of IPV

One quarter of respondents reported experiencing any IPV in their current relationship (24.5%; Table 3). Sexual violence was the most prevalent domain, reported by 16.0% of respondents, followed by economic control (12.5%). Fewer participants reported psychological (6.0%) or physical (3.5%) violence.

### Relationship Between ACEs, IPV and Depressive Symptoms

ACEs score was positively and significantly associated with severity of IPV after controlling for sociodemographic covariates. Each ACE reported was associated with a mean 0.23-point (95% CI [0.20, 0.36]) increase in IPV severity score. Relationship quality was independently associated with IPV; compared with participants endorsing the highest relationship quality, those endorsing only a high relationship quality had on average 1.52 point higher IPV severity score (95% CI [0.69, 2.35]). Household wealth had a marginally significant influence on severity of IPV in this model.

Both ACEs score and IPV were found to be significantly associated with increased depressive symptoms. Each ACEs reported was associated with a mean 1.92-point increase in depressive symptom score (95% CI [1.11, 2.73]), and, in a separate model, having experienced any ACEs was associated with over two-fold odds of depression (Odds ratio 2.10, 95% CI [1.05, 4.23], *p* = .037); not shown). Each one-point increase in IPV score was associated with a mean 1.25-point (95% CI [0.45, 2.06]) increase in depressive symptoms. Relationship quality was independently associated with depressive symptoms; compared with participants endorsing the highest relationship quality, those endorsing only a high relationship quality reported on average a 6.26-point higher level of depressive symptoms (95% CI [1.11, 2.73]). Rural residence was associated with a mean 3.84-point (95% CI [−6.52, −1.17]) lower level of depressive symptoms. No other sociodemographic characteristics were independently associated with level of depressive symptoms.

In decomposition analyses of the effect of ACEs score on depressive symptoms (Table 4), the indirect pathway was not statistically significant, indicating no significant mediation of ACEs exposure through IPV, yet significant direct effects of both ACEs exposure and IPV on depression.

**Table 4.** Relationships Between ACEs, Intimate Partner Violence and Depressive Symptoms, Newly Married Women Study Participants, Nawalparasi, Nepal, 2018 (*N* = 200).

Predictors	β	95% CI	*p*
Outcome: Depressive symptoms score			
IPV score	1.25	0.45, 2.06	.002
ACEs score	1.92	1.11, 2.73	<.001
Relationship quality with husband			
High	6.26	1.30, 11.21	.013
Highest	REF	—	—
Age group			
18–20	REF	—	—
21–22	−2.08	−4.66, 0.50	.114
23+	−2.42	−6.83, 0.98	.163
Educational attainment			
None	−0.13	−5.95, 5.69	.909
Primary (1–8)	−1.40	−4.25, 1.45	.335
Secondary (9–12)	REF	—	—
>12	−0.08	−3.76, 3.60	.966
Wealth quintile			
Lowest	REF	—	—
Second	0.59	−2.91, 4.10	.739
Middle	−0.65	−4.26, 2.95	.723
Fourth	−1.92	−5.89, 2.05	.344
Highest	−3.31	−6.49, −1.21	.145
Rurality			
Urban	REF	—	—
Rural	−3.85	−6.49, −1.21	.004
Outcome: Intimate partner violence severity score			
ACEs score	0.23	0.20, 0.36	.001
Relationship quality with husband			
High	1.52	0.69, 2.35	<.001
Highest	REF	—	—
Age group			
18–20	REF	—	—
21–22	−0.34	−0.78, 0.10	.134
23+	−0.42	−1.00, 0.17	.176
Outcome: Intimate partner violence severity score			
Educational attainment			
None	0.53	−0.47, 1.53	.302
Primary (1–8)	−0.17	−0.66, 0.32	.489
Secondary (9–12)	REF	—	—
More than secondary (>12)	−0.21	−0.84, 0.42	.513
Wealth quintile			
Lowest	REF	—	—
Second	−0.49	−1.09, 0.11	.113
Middle	−0.43	−1.05, 0.19	.171
Fourth	−0.62	−1.30, 0.06	.074
Highest	−0.31	−0.72, 0.19	.253
Rurality			
Urban	REF	—	—
Rural	−0.26	−0.72, 0.19	.253
Effect decomposition, mediation analysis			
Indirect	0.29	−1.15, 0.72	.197
Direct	1.92	0.94, 2.91	<.001
Total	2.21	1.21, 3.21	<.001

*Note*. CI = confidence interval; ACE = adverse childhood experience.

## Discussion

The findings from our study contribute to the literature on ACEs, marital relationships, and mental health among young, newly married Nepalese women in several ways. First, the high prevalence of depressive symptoms and even higher prevalence of ACEs identified among our study sample confirm the critical importance of further research and intervention among this socially vulnerable population to improve mental health morbidity and reduce adverse experiences. Furthermore, the positive association observed between ACEs and depressive symptoms, the identification of IPV as an independent contributor to depressive symptoms but not a significant mediator, and the important role of relationship quality provide important information on the unique pathways through which distal and proximate factors such as these influence mental health status.

More than half (58%) of young, newly married women in our sample experienced at least one ACE, most of which were within the parental presence, emotional abuse and neglect, and household violence domains. While this represents a large proportion of our sample, the prevalence is somewhat lower than other ACEs rates identified from research among young adult females in other lower income settings, which range from 80% among a nationally representative sample of 18 to 24 year old women in Honduras, to 85% to 88% among similarly aged women in urban South Africa, Brazil, and Kashmiri university students from both urban and rural settings (Dar et al., 2022; Kappel et al., 2021; Manyema & Richter, 2019; Soares et al., 2016). Differences between our sample and these studies may be partially due to some differences in rural versus urban status, and socioeconomic status, which varied among the studies though reflects lower quintiles in our study. Nationally representative studies from higher resourced settings report lower ACEs prevalences but evidence large gradients by social exposures such as region, poverty status, race, and ethnicity (Haahr-Pedersen et al., 2020).

Consistent with other research on young adults globally (Al Shawi et al., 2019; Blum et al., 2019; Chapman et al., 2004; Iob et al., 2022), we found ACEs score to be significantly and positively associated with depressive symptom severity and odds of depression. Our findings underscore the critical and enduring role of childhood exposures on young adult mental health. Furthermore, these findings contribute to the expanding evidence base demonstrating a high burden of mental health morbidity in South Asian countries and calling for further research and intervention implementation in this context (Naveed et al., 2020).

While the parent study was not designed to evaluate a comprehensive array of potential multilevel social and biological contributors to depressive symptoms among our study participants and thus was limited in its analysis, the current analysis did disentangle the role of IPV on depression, identifying that IPV operated independently of ACEs exposure on increasing risk of depressive symptoms, and confirming the importance of both of these exposures for mental health. Prior literature supports a strong influence of ACEs on both IPV victimization and perpetration (Zhu et al., 2023). Given the prevalence of IPV reported by women of reproductive age in this setting, particularly new mothers (Dalal et al., 2014; Raifman et al., 2021; Subba et al., 2013), targeted interventions to mitigate both ACEs and IPV are warranted and are likely to not only improve the health and well-being of these individuals, but interrupt cycles of intergenerational adversity (Craig et al., 2021; Narayan et al., 2021).

Expanding the research on ACEs, resilience, and health outcomes across varied contexts is important for understanding multilevel opportunities for mitigation. Newly married women living in Nepal are living within a unique social context characterized by gender inequity and patriarchy, where most marriages are arranged and most women move into the husband’s household after marriage to co-reside with their in-laws. In the marital household, new brides occupy the lowest status in the household, which has tangible physical and emotional impacts (Gopalakrishnan et al., 2023b).

### Strengths and Limitations

This study adds to the literature on ACEs and mental health through expanding our characterization of these relationships into the unique under-researched population sample of young, newly married women in rural Nepal. To our knowledge, this is this first paper reporting on ACEs and relationship to early adult mental health and IPV among this population in Nepal. This population has married only recently and is co-residing with in-laws in a new household, many within a new community. Findings should be interpreted while considering certain limitations including potential lack of generalizability due to our focus on one region of Nepal, the sociodemographic profiles of our study participants (e.g. young adult females, largely with secondary education completed, majority Hindu, and relatively low socioeconomic status), the cross-sectional design of this study and lack of longitudinal and comprehensive data including women’s natal home characteristics, their prior mental health status, social support, and other factors influencing resilience, family history of mental health morbidity, or other health risk behaviors. Future research on this topic in this setting would be improved by the assessment of the role of these factors and a more complete picture would be identified through using a dyadic approach given the newly married nature of these women, and the important role of IPV and relationship quality. Incorporation of attachment style measures would also benefit future studies, allowing alignment with other recent research identifying the important role of attachment type in IPV victimization (Stefania et al., 2023). Finally, as with all questions asked about past experiences, our evaluation of ACEs may be subject to recall bias.

### Conclusions

Applying a gender lens to understanding the impact of ACEs on mental health in a lower resource context is critical given the gap in this literature and the importance of these factors for health across the lifecourse. These findings suggest that ACEs are an important influencer of young adult poor mental health. Policies and programs needed to prevent and mitigate the impact of ACEs, and given the potential impact of protective factors, targeting the marital home and transition as a time to build safe spaces and support for women should be considered. Additionally, preventing violence of all forms experienced by women in childhood and into early adulthood will have lasting impacts on their mental and physical health, and likely on future generations as well.

## Supplemental Material

sj-docx-1-jiv-10.1177_08862605261446978 – Supplemental material for Adverse Childhood Events are Associated with Poor Mental Health in Young, Newly Married Women in Nepal Supplemental material, sj-docx-1-jiv-10.1177_08862605261446978 for Adverse Childhood Events are Associated with Poor Mental Health in Young, Newly Married Women in Nepal by Alison M. El Ayadi, Saira Khan, Mahesh C. Puri and Nadia G. Diamond-Smith in Journal of Interpersonal Violence

sj-docx-2-jiv-10.1177_08862605261446978 – Supplemental material for Adverse Childhood Events are Associated with Poor Mental Health in Young, Newly Married Women in Nepal Supplemental material, sj-docx-2-jiv-10.1177_08862605261446978 for Adverse Childhood Events are Associated with Poor Mental Health in Young, Newly Married Women in Nepal by Alison M. El Ayadi, Saira Khan, Mahesh C. Puri and Nadia G. Diamond-Smith in Journal of Interpersonal Violence
