# Supplementary material for: Adverse Childhood Events are Associated with Poor Mental Health in Young, Newly Married Women in Nepal
Source: J Interpers Violence. 2026 May 31;41(19-20):7558–82. doi: 10.1177/08862605261446978 (PMC13570970; doi:10.1177/08862605261446978)
Supplement: Supplementary file 1 [file sj-docx-1-jiv-10.1177_08862605261446978.docx]

**Table S1a. Exposure to adverse childhood experiences (ACEs) by sociodemographic characteristics among newly married women study participants, Nawalparasi, Nepal, 2018 (n=200)**

|  | **Any ACEs** | | | |  | **Parental Presence** | | | |  | **Household Violence** | | | |  |
| --- | --- | --- | --- | --- | --- | --- | --- | --- | --- | --- | --- | --- | --- | --- | --- |
|  | **Yes** | | **No** | | **P** | **Yes** | | **No** | | **P** | **Yes** | | **No** | | **P** |
|  | **N** | **%** | **N** | **%** |  | **N** | **%** | **N** | **%** |  | **N** | **%** | **N** | **%** |  |
| Age at Last Birthday | 20.4 (2.0) | | 20.5 (2.0) | | 0.715 | 20.3 (1.9) | | 20.5 (1.0) | | 0.539 | 20.3 (2.1) | | 20.5 (2.0) | | 0.574 |
| Age When First Married | 20.4 (2.0) | | 20.5 (2.0) | | 0.621 | 10.3 (2.0) | | 20.5 (2.0) | | 0.437 | 20.2 (2.2) | | 20.5 (2.0) | | 0.380 |
| Marriage Type (Love or Arranged) |  |  |  |  | 0.001 |  |  |  |  | 0.002 |  |  |  |  | 0.709 |
| Arranged | 72 | 51.1 | 69 | 48.9 |  | 43 | 30.5 | 98 | 69.5 |  | 37 | 26.2 | 104 | 73.8 |  |
| Love | 45 | 76.3 | 14 | 23.7 |  | 32 | 54.2 | 27 | 45.8 |  | 17 | 28.8 | 42 | 71.2 |  |
| Religion |  |  |  |  | 0.167 |  |  |  |  | 0.169 |  |  |  |  | 0.710 |
| Hindu | 100 | 58.1 | 72 | 41.9 |  | 66 | 38.4 | 106 | 61.6 |  | 49 | 28.5 | 123 | 71.5 |  |
| Buddhist | 8 | 88.9 | 1 | 11.1 |  | 5 | 55.6 | 4 | 44.4 |  | 1 | 11.1 | 8 | 88.9 |  |
| Muslim | 8 | 47.1 | 9 | 52.9 |  | 3 | 17.6 | 14 | 82.4 |  | 4 | 23.5 | 13 | 76.5 |  |
| Bon | 1 | 100.0 | 0 | 0.0 |  | 1 | 100.0 | 0 | 0.0 |  | 0 | 0.0 | 1 | 100.0 |  |
| Christian | 0 | 0.0 | 1 | 100.0 |  | 0 | 0.0 | 1 | 100.0 |  | 0 | 0.0 | 1 | 100.0 |  |
| Caste |  |  |  |  | 0.008 |  |  |  |  | 0.017 |  |  |  |  | 0.150 |
| Brahmin | 11 | 36.7 | 19 | 63.3 |  | 6 | 20.0 | 24 | 80.0 |  | 3 | 10.0 | 27 | 90.0 |  |
| Chhetri | 5 | 45.5 | 6 | 54.5 |  | 2 | 18.2 | 9 | 81.8 |  | 2 | 18.2 | 9 | 81.8 |  |
| Tarai Dalit | 12 | 66.7 | 6 | 33.3 |  | 8 | 44.4 | 10 | 55.6 |  | 5 | 27.8 | 13 | 72.2 |  |
| Janajati | 21 | 87.5 | 3 | 12.5 |  | 12 | 50.0 | 12 | 50.0 |  | 11 | 45.8 | 13 | 54.2 |  |
| Terai Janjati | 44 | 56.4 | 34 | 43.6 |  | 30 | 38.5 | 48 | 61.5 |  | 23 | 29.5 | 55 | 70.5 |  |
| Muslim | 8 | 50.0 | 8 | 50.0 |  | 3 | 18.8 | 13 | 81.3 |  | 4 | 25.0 | 12 | 75.0 |  |
| Other | 16 | 69.6 | 7 | 30.4 |  | 14 | 60.9 | 9 | 39.1 |  | 6 | 26.1 | 17 | 73.9 |  |
| Has Been Pregnant Before |  |  |  |  | 0.037 |  |  |  |  | 0.058 |  |  |  |  | 0.051 |
| No | 103 | 56.3 | 80 | 43.7 |  | 65 | 35.5 | 118 | 64.5 |  | 46 | 25.1 | 137 | 74.9 |  |
| Yes | 14 | 82.4 | 3 | 17.6 |  | 10 | 58.8 | 7 | 41.2 |  | 8 | 47.1 | 9 | 52.9 |  |
| Has Toilet and Running Water |  |  |  |  | 0.849 |  |  |  |  | 0.805 |  |  |  |  | 0.355 |
| No | 86 | 58.9 | 60 | 41.1 |  | 54 | 37.0 | 92 | 63.0 |  | 42 | 28.8 | 104 | 71.2 |  |
| Yes | 31 | 57.4 | 23 | 42.6 |  | 21 | 38.9 | 33 | 61.1 |  | 12 | 22.2 | 42 | 77.8 |  |
| Has Electricity |  |  |  |  | 0.234 |  |  |  |  | 0.437 |  |  |  |  | 0.542 |
| No | 0 | 0.0 | 1 | 100.0 |  | 0 | 0.0 | 1 | 100.0 |  | 0 | 0.0 | 1 | 100.0 |  |
| Yes | 117 | 58.8 | 82 | 41.2 |  | 75 | 37.7 | 124 | 62.3 |  | 54 | 27.1 | 145 | 72.9 |  |
| Household Fuel |  |  |  |  | 0.962 |  |  |  |  | 0.697 |  |  |  |  | 0.854 |
| LPG Gas | 58 | 58.6 | 41 | 41.4 |  | 40 | 40.4 | 59 | 59.6 |  | 25 | 25.3 | 74 | 74.7 |  |
| Firewood | 53 | 58.9 | 37 | 41.1 |  | 31 | 34.4 | 59 | 65.6 |  | 26 | 28.9 | 64 | 71.1 |  |
| Other | 6 | 54.5 | 5 | 45.5 |  | 4 | 36.4 | 7 | 63.6 |  | 3 | 27.3 | 8 | 72.7 |  |
| Parents Living in Same Town/Village |  |  |  |  | 0.700 |  |  |  |  | 0.222 |  |  |  |  | 0.040 |
| No | 99 | 58.2 | 71 | 41.8 |  | 66 | 38.8 | 104 | 61.2 |  | 41 | 24.1 | 129 | 75.9 |  |
| Yes | 17 | 58.6 | 12 | 41.4 |  | 8 | 27.6 | 21 | 72.4 |  | 12 | 41.4 | 17 | 58.6 |  |
| N/A | 1 | 100.0 | 0 | 0.0 |  | 1 | 100.0 | 0 | 0.0 |  | 1 | 100.0 | 0 | 0.0 |  |
| Educational attainment |  |  |  |  | 0.235 |  |  |  |  | 0.225 |  |  |  |  | 0.054 |
| None | 4 | 50.0 | 4 | 50.0 |  | 3 | 37.5 | 5 | 62.5 |  | 1 | 12.5 | 7 | 87.5 |  |
| Primary 1-8 | 37 | 68.5 | 17 | 31.5 |  | 25 | 46.3 | 29 | 53.7 |  | 21 | 38.9 | 33 | 61.1 |  |
| Secondary 9-12 | 62 | 57.4 | 46 | 42.6 |  | 40 | 37.0 | 68 | 63.0 |  | 28 | 25.9 | 80 | 74.1 |  |
| Higher than secondary | 14 | 46.7 | 16 | 53.3 |  | 7 | 23.3 | 23 | 76.7 |  | 4 | 13.3 | 26 | 86.7 |  |
| Number of Months Married | 7.6 (1.1) | | 7.7 (1.1) | | 0.655 | 7.5 (1.1) | | 7.7 (1.1) | | 0.392 | 7.6 (1.0) | | 7.6 (1.1) | | 0.145 |
| Mother-in-Law Relationship Quality |  |  |  |  | 0.423 |  |  |  |  | 0.664 |  |  |  |  | 0.056 |
| Lowest | 0 | 0.0 | 3 | 100.0 |  | 2 | 66.7 | 1 | 33.3 |  | 2 | 66.7 | 1 | 33.3 |  |
| Low | 4 | 36.4 | 7 | 63.6 |  | 5 | 45.5 | 6 | 54.5 |  | 6 | 54.5 | 5 | 45.5 |  |
| High | 44 | 40.4 | 65 | 59.6 |  | 41 | 37.6 | 68 | 62.4 |  | 25 | 22.9 | 84 | 77.1 |  |
| Highest | 35 | 45.5 | 42 | 54.5 |  | 27 | 35.1 | 50 | 64.9 |  | 21 | 27.3 | 56 | 72.7 |  |
| Spousal Relationship Quality |  |  |  |  | 0.366 |  |  |  |  | 0.936 |  |  |  |  | 0.983 |
| High | 5 | 45.5 | 6 | 54.5 |  | 4 | 36.4 | 7 | 63.6 |  | 3 | 27.3 | 8 | 72.7 |  |
| Highest | 112 | 59.3 | 77 | 40.7 |  | 71 | 37.6 | 118 | 62.4 |  | 51 | 27.0 | 138 | 73.0 |  |
