# Supplementary material for: Adverse Childhood Events are Associated with Poor Mental Health in Young, Newly Married Women in Nepal
Source: J Interpers Violence. 2026 May 31;41(19-20):7558–82. doi: 10.1177/08862605261446978 (PMC13570970; doi:10.1177/08862605261446978)
Supplement: Supplementary file 2 [file sj-docx-2-jiv-10.1177_08862605261446978.docx]

**Table S1b. Exposure to adverse childhood experiences (ACEs) by sociodemographic characteristics among newly married women study participants, Nawalparasi, Nepal, 2018 (n=200)**

|  | **Emot. Abuse/Neglect** | | | |  | **Phys Abuse** | | | |  | **Sexual Abuse** | | | |  |
| --- | --- | --- | --- | --- | --- | --- | --- | --- | --- | --- | --- | --- | --- | --- | --- |
|  | **Yes** | | **No** | | **P** | **Yes** | | **No** | | **P** | **Yes** | | **No** | | **P** |
|  | **N** | **%** | **N** | **%** |  | **N** | **%** | **N** | **%** |  | **N** | **%** | **N** | **%** |  |
| Age at Last Birthday | 20.1 (2.0) | | 10.6 (2.0) | | 0.145 | 19.6 (1.5) | | 20.5 (2.0) | | 0.033 | 18.3 (0.5) | | 20.5 (2.0) | | 0.025 |
| Age When First Married | 20.1 (2.1) | | 20.6 (2.0) | | 0.099 | 19.6 (1.5) | | 20.5 (2.0) | | 0.044 | 18.3 (0.5) | | 20.5 (2.0) | | 0.030 |
| Marriage Type | | |  |  | 0.001 |  |  |  |  | 0.034 |  |  |  |  | 0.364 |
| Arranged | 34 | 24.1 | 107 | 75.9 |  | 10 | 7.1 | 131 | 92.9 |  | 2 | 1.4 | 139 | 98.6 |  |
| Love | 29 | 49.2 | 30 | 50.8 |  | 10 | 16.9 | 49 | 83.1 |  | 2 | 3.4 | 57 | 96.6 |  |
| Religion |  |  |  |  | 0.429 |  |  |  |  | 0.193 |  |  |  |  | 0.956 |
| Hindu | 54 | 31.4 | 118 | 68.6 |  | 16 | 9.3 | 156 | 90.7 |  | 4 | 2.3 | 168 | 97.7 |  |
| Buddhist | 5 | 55.6 | 4 | 44.4 |  | 3 | 33.3 | 6 | 66.7 |  | 0 | 0.0 | 9 | 100.0 |  |
| Muslim | 4 | 23.5 | 13 | 76.5 |  | 1 | 5.9 | 16 | 94.1 |  | 0 | 0.0 | 17 | 100.0 |  |
| Bon | 0 | 0.0 | 1 | 100.0 |  | 0 | 0.0 | 1 | 100.0 |  | 0 | 0.0 | 1 | 100.0 |  |
| Christian | 0 | 0.0 | 1 | 100.0 |  | 0 | 0.0 | 1 | 100.0 |  | 0 | 0.0 | 1 | 100.0 |  |
| Caste |  |  |  |  | 0.005 |  |  |  |  | 0.036 |  |  |  |  | 0.710 |
| Brahmin | 5 | 16.7 | 25 | 83.3 |  | 1 | 3.3 | 29 | 96.7 |  | 0 | 0.0 | 30 | 100.0 |  |
| Chhetri | 4 | 36.4 | 7 | 63.6 |  | 1 | 9.1 | 10 | 90.9 |  | 0 | 0.0 | 11 | 100.0 |  |
| Tarai Dalit | 9 | 50.0 | 9 | 50.0 |  | 5 | 27.8 | 13 | 72.2 |  | 1 | 5.6 | 17 | 94.4 |  |
| Janajati | 15 | 62.5 | 9 | 37.5 |  | 5 | 20.8 | 19 | 79.2 |  | 1 | 4.2 | 23 | 95.8 |  |
| Terai Janjati | 20 | 25.6 | 58 | 74.4 |  | 4 | 5.1 | 74 | 94.9 |  | 1 | 1.3 | 77 | 98.7 |  |
| Muslim | 4 | 25.0 | 12 | 75.0 |  | 1 | 6.3 | 15 | 93.8 |  | 0 | 0.0 | 16 | 100.0 |  |
| Other | 6 | 26.1 | 17 | 73.9 |  | 3 | 13.0 | 20 | 87.0 |  | 1 | 4.3 | 22 | 95.7 |  |
| Has Been Pregnant Before | |  |  |  | 0.047 |  |  |  |  | 0.052 |  |  |  |  | 0.232 |
| No | 54 | 29.5 | 129 | 70.5 |  | 16 | 8.7 | 167 | 91.3 |  | 3 | 1.6 | 180 | 98.4 |  |
| Yes | 9 | 52.9 | 8 | 47.1 |  | 4 | 23.5 | 13 | 76.5 |  | 1 | 5.9 | 16 | 94.1 |  |
| Has Toilet and Running Water | | |  |  | 0.997 |  |  |  |  | 0.396 |  |  |  |  | 0.295 |
| No | 46 | 31.5 | 100 | 68.5 |  | 13 | 8.9 | 133 | 91.1 |  | 2 | 1.4 | 144 | 98.6 |  |
| Yes | 17 | 31.5 | 37 | 68.5 |  | 7 | 13.0 | 47 | 87.0 |  | 2 | 3.7 | 52 | 96.3 |  |
| Has Electricity |  |  |  |  | 0.497 |  |  |  |  | 0.738 |  |  |  |  | 0.886 |
| No | 0 | 0.0 | 1 | 100.0 |  | 0 | 0.0 | 1 | 100.0 |  | 0 | 0.0 | 1 | 100.0 |  |
| Yes | 63 | 31.7 | 136 | 68.3 |  | 20 | 10.1 | 179 | 89.9 |  | 4 | 2.0 | 195 | 98.0 |  |
| Household Fuel |  |  |  |  | 0.938 |  |  |  |  | 0.126 |  |  |  |  | 0.884 |
| LPG Gas | 31 | 31.3 | 68 | 68.7 |  | 10 | 10.1 | 89 | 89.9 |  | 2 | 2.0 | 97 | 98.0 |  |
| Firewood | 28 | 31.1 | 62 | 68.9 |  | 7 | 7.8 | 83 | 92.2 |  | 2 | 2.2 | 88 | 97.8 |  |
| Other | 4 | 36.4 | 7 | 63.6 |  | 3 | 27.3 | 8 | 72.7 |  | 0 | 0.0 | 11 | 100.0 |  |
| Parents Living in Same Town/Village | | |  |  | 0.077 |  |  |  |  | 0.008 |  |  |  |  | 0.698 |
| No | 49 | 28.8 | 121 | 71.2 |  | 15 | 8.8 | 155 | 91.2 |  | 4 | 2.4 | 166 | 97.6 |  |
| Yes | 13 | 44.8 | 16 | 55.2 |  | 4 | 13.8 | 25 | 86.2 |  | 0 | 0.0 | 29 | 100.0 |  |
| No parents (passed away) | 1 | 100.0 | 0 | 0.0 |  | 1 | 100.0 | 0 | 0.0 |  | 0 | 0.0 | 1 | 100.0 |  |
| Educational attainment | |  |  |  | 0.246 |  |  |  |  | 0.438 |  |  |  |  | 0.324 |
| None | 2 | 25.0 | 6 | 75.0 |  | 0 | 0.0 | 8 | 100.0 |  | 0 | 0.0 | 8 | 100.0 |  |
| Primary 1-8 | 20 | 37.0 | 34 | 63.0 |  | 8 | 14.8 | 46 | 85.2 |  | 0 | 0.0 | 54 | 100.0 |  |
| Secondary 9-12 | 36 | 33.3 | 72 | 66.7 |  | 10 | 9.3 | 98 | 90.7 |  | 4 | 3.7 | 104 | 96.3 |  |
| Higher than 2 | 5 | 16.7 | 25 | 83.3 |  | 2 | 6.7 | 28 | 93.3 |  | 0 | 0.0 | 30 | 100.0 |  |
| Number of Months Married | 7.5 (1.1) | | 7.7 (1.1) | | 0.327 | 7.3 (1.1) | | 7.7 (1.1) | | 0.106 | 7.1 (1.3) | | 7.6 (1.1) | | 0.248 |
| Mother-in-Law Relationship Quality | | |  |  | 0.073 |  |  |  |  | 0.034 |  |  |  |  | <0.001 |
| Lowest | 3 | 100.0 | 0 | 0.0 |  | 1 | 33.3 | 2 | 66.7 |  | 1 | 33.3 | 2 | 66.7 |  |
| Low | 4 | 36.4 | 7 | 63.6 |  | 3 | 27.3 | 8 | 72.7 |  | 1 | 9.1 | 10 | 90.9 |  |
| High | 34 | 31.2 | 75 | 68.8 |  | 6 | 5.5 | 103 | 94.5 |  | 2 | 1.8 | 107 | 98.2 |  |
| Highest | 22 | 28.6 | 55 | 71.4 |  | 10 | 13.0 | 67 | 87.0 |  | 0 | 0.0 | 77 | 100.0 |  |
| Spousal Relationship Quality | |  |  |  | 0.721 |  |  |  |  | 0.352 |  |  |  |  | 0.626 |
| High | 4 | 36.4 | 7 | 63.6 |  | 2 | 18.2 | 9 | 81.8 |  | 0 | 0.0 | 11 | 100.0 |  |
| Highest | 59 | 31.2 | 130 | 68.8 |  | 18 | 9.5 | 171 | 90.5 |  | 4 | 2.1 | 185 | 97.9 |  |
